# Seasonal variations of soil bacterial and fungal communities in a subtropical *Eucalyptus* plantation and their responses to throughfall reduction

**DOI:** 10.3389/fmicb.2023.1113616

**Published:** 2023-03-28

**Authors:** Yubiao Lin, Ling Yang, Zetao Chen, Yaqian Gao, Jiejun Kong, Qian He, Yan Su, Jiyue Li, Quan Qiu

**Affiliations:** Guangdong Key Laboratory for Innovative Development and Utilization of Forest Plant Germplasm, College of Forestry and Landscape Architecture, South China Agricultural University, Guangzhou, China

**Keywords:** *Eucalyptus* plantation, seasonal variations, microbial diversity, microbial functions, co-occurrence networks

## Abstract

Climatic change causes obvious seasonal meteorological drought in southern China, yet there is a lack of comprehensive in situ studies on the effects of drought in *Eucalyptus* plantations. Here, a 50% throughfall reduction (TR) experiment was conducted to investigate the seasonal variations of soil bacterial and fungal communities and functions in a subtropical *Eucalyptus* plantation and their responses to TR treatment. Soil samples were collected from control (CK) and TR plots in the dry and rainy seasons and were subjected to high-throughput sequencing analysis. Results showed that TR treatment significantly reduced soil water content (SWC) in the rainy season. In CK and TR treatments, fungal alpha-diversity decreased in the rainy season while bacterial alpha-diversity did not change significantly between dry and rainy seasons. Moreover, bacterial networks were more affected by seasonal variations compared with fungal networks. Redundancy analysis showed that alkali hydrolyzed nitrogen and SWC contributed the most to the bacterial and fungal communities, respectively. Functional prediction indicated that the expression of soil bacterial metabolic functions and symbiotic fungi decreased in the rainy season. In conclusion, seasonal variations have a stronger effect on soil microbial community composition, diversity, and function compared with TR treatment. These findings could be used to develop management practices for subtropical *Eucalyptus* plantations and help maintain soil microbial diversity to sustain long-term ecosystem function and services in response to future changes in precipitation patterns.

## Introduction

Changes in precipitation patterns and an increase in global mean air temperatures are causing drought stress in many parts of the world (Jiang and Wang, 2021). Global warming is predicted to lead to greater drought frequency in China, which will subsequently lead to more frequent seasonal variation in soil moisture (Chen and Yuan, 2021). Long-term droughts and heavy rains cause quick changes in soil moisture, which affect the development and activity of microorganisms and have an impact on the cycling of soil carbon (Schimel, 2018). Although numerous studies have evaluated howclimate change is affecting soil microbial communities (Bahram et al., 2018; Zhang et al., 2022), the results are largely inconsistent and very few studies have concentrated on seasonal variations in soil microbial communities and the reactions to reduced precipitation (Jurburg et al., 2018; Agoussar et al., 2021; Azarbad et al., 2022).

Soil microorganisms are an essential part of the forest ecosystem and are crucial to many fundamental ecological processes, particularly the decomposition of soil organic matter and the cycling of nutrients in forest ecosystems (Jiao et al., 2018; Wagg et al., 2019). Soil microorganisms are influenced by many environmental factors. For example, soil microorganisms are very sensitive to changes in moisture and temperature conditions; and increased in moisture, sustained warming, and earlier drought times can increase soil microbial C and N (Li et al., 2015; Wang et al., 2015; Gao et al., 2018). Seasonal precipitation significantly increases soil rare bacteria and dominant fungi (He et al., 2017; Zhao et al., 2017). Additionally, the composition and function of soil microbial communities are impacted by the availability of soil water (Brockett et al., 2012; Diaz-Pereira et al., 2019); however, the direction and magnitude of soil microbial community composition and function following changes in precipitation remain uncertain (Zhao et al., 2017; He et al., 2022). Furthermore, there is a lack of studies investigating the soil microbial community network structure in response to precipitation change (He et al., 2017).

Microorganisms thrive in communities where they can interact closely and generate increased benefits for the community as a whole, rather than living in isolation (Faust and Raes, 2012; Röttjers and Faust, 2018). Soil microorganisms often interact in complex interspecific networks, which can affect the microbial community’s response to climate change (De Vries et al., 2018). In complex microbial communities, network analysis has proven to be a powerful way to explore the interactions between different taxa (Barberán et al., 2012; Wang et al., 2018). Despite its shortcomings (Faust and Raes, 2012), network analysis offers crucial new understandings for the structure of microbial communities, the relationships between taxa, and how these taxa react to environmental change. It might be more challenging to comprehend using conventional indicators of diversity that are often employed in microbial ecology (Lu et al., 2013).

In southern China, *Eucalyptus* is widely planted across an area of 5.46 million hectares, accounting for 2.5% of country’s total forest area and contributing to more than one-third of the total timber production (Arnold et al., 2020). Consequently, it is crucial to study how climate change is affecting the *Eucalyptus* plantation ecosystem. However, seasonal variations of soil microbial communities (bacterial and fungi) in subtropical *Eucalyptus* plantations and their responses to reduced precipitation have not yet been reported. Therefore, we investigated the seasonal variations in the composition and predicted function of soil bacterial and fungal communities and their responses to throughfall reduction (TR) by setting up a fixed throughfall reduction sample plot in a subtropical *Eucalyptus* plantation. We hypothesized that (I) seasonal variations have a more significant effect on diversity, composition, networks and functional predictions of soil microbial communities, compared with TR treatment; (II) seasonal variations have a more significant effect on soil bacterial and fungal communities compared with TR treatment; (III) bacterial taxonomical composition and networks are more influenced by seasonal variations compared with fungal taxonomical composition and networks.

## Materials and methods

### Study site

**Figure 1:**
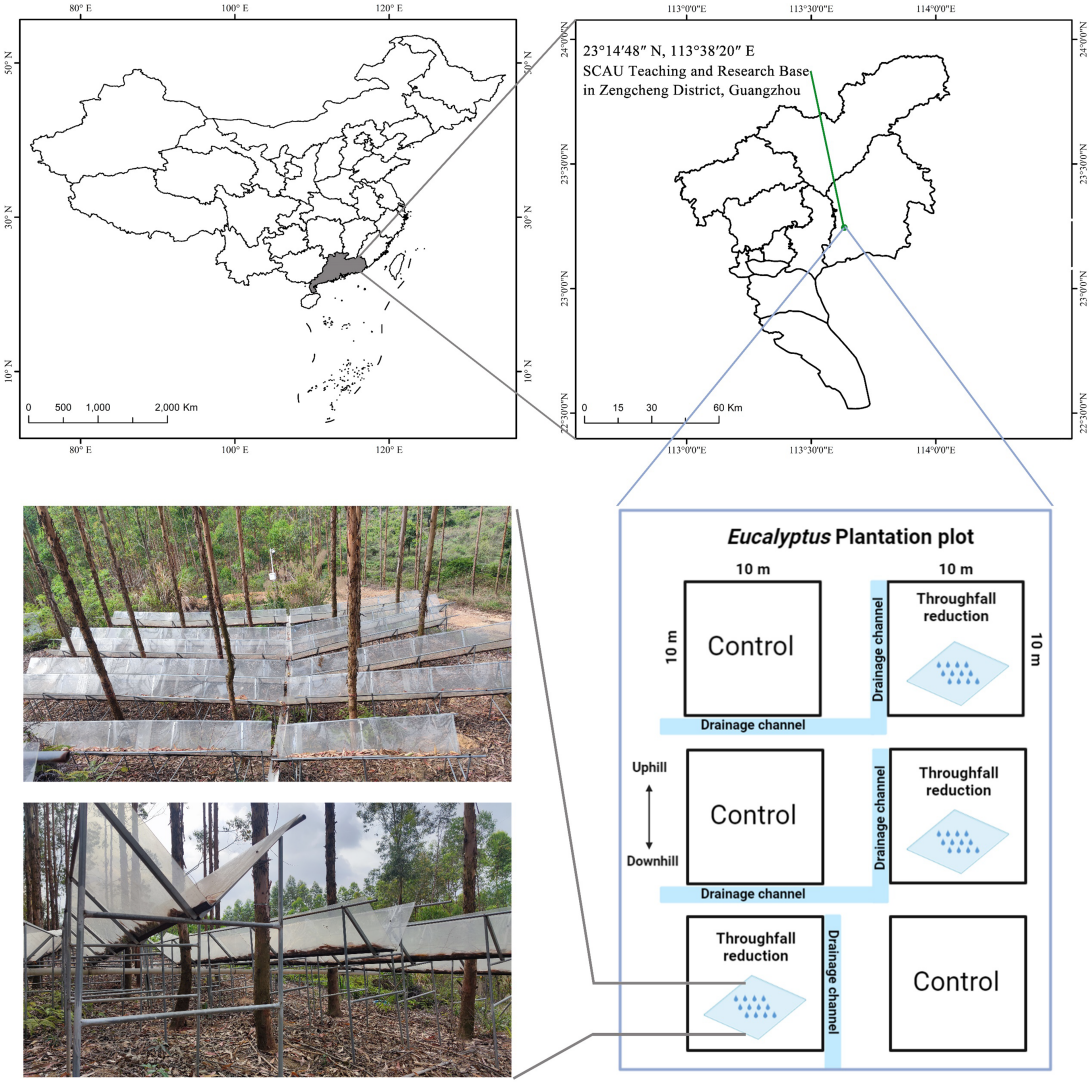
Study area and sampling sites in the Zengcheng district, Guangdong Province of China.

**Figure 2:**
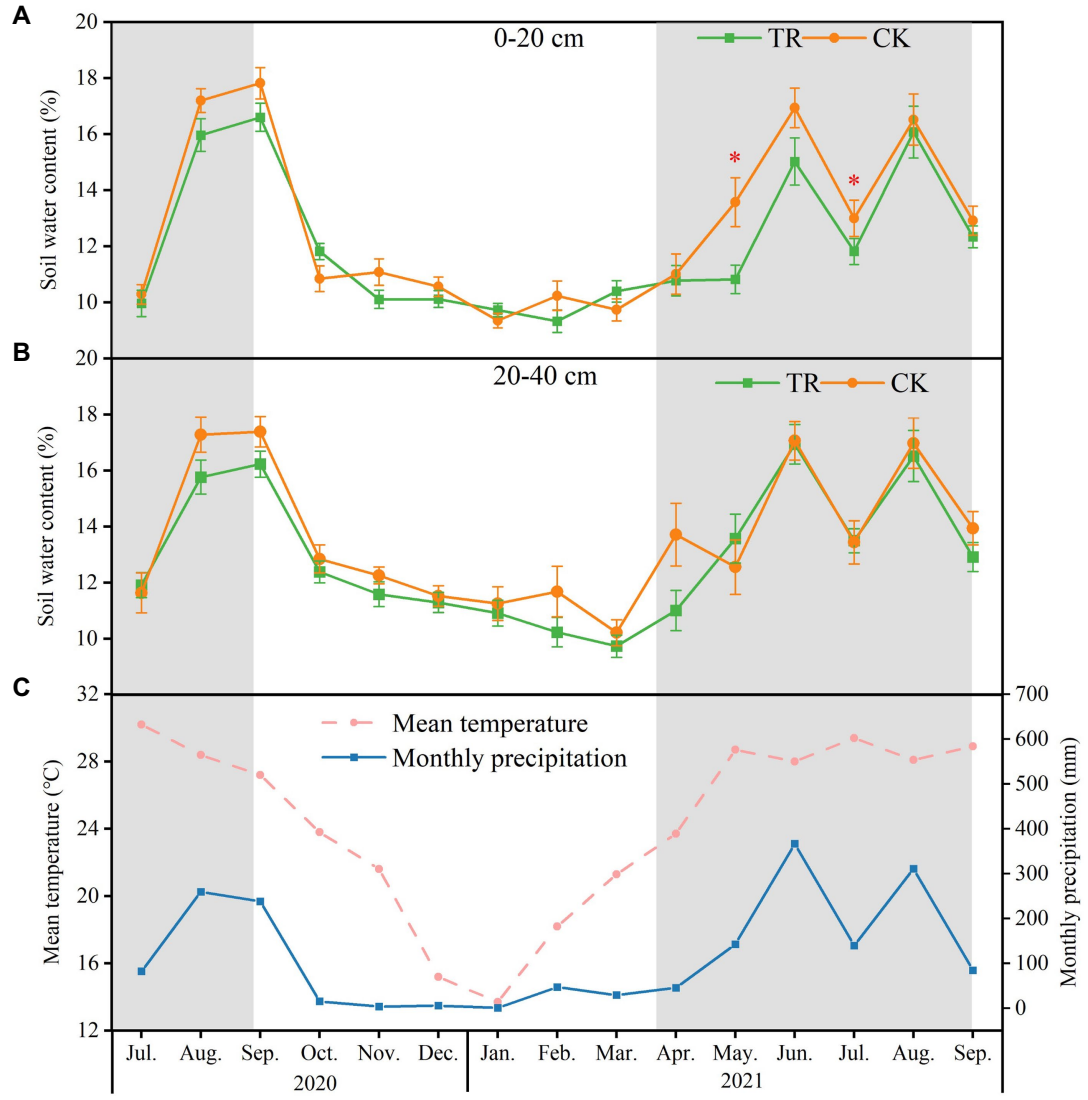
Soil water content at 0–20 cm **(A)** and 20–40 cm **(B)**, and monthly precipitation and mean temperature **(C)** from July 2020 to September 2021. Values are means + standard error (*n* = 9). CK: control; TR: throughfall reduction. Asterisks represent a significant difference (*p* < 0.05) in the Kruskal–Wallis test.

The study site was located in South China Agricultural University (SCAU) Teaching & Research Base in Zengcheng District, Guangzhou, China (23°14′48″ N, 113°38′20″ E) (Figure 1). South subtropical monsoon climate prevails in this area, with a dry season lasting from October to March and a rainy season spanning from April to September. It has a mean annual temperature of 21.9°C, and mean annual precipitation of 2004.5 mm, with about 80% occurring during the rainy season (Figure 2C).

### Experimental design

A block experimental design was employed, and the study area consisted of six experimental plots, including three TR treatment plots and three control plots, each measuring 10 m × 10 m. For each TR plot, steel frames were mounted on standing steel posts as shelters to reduce throughfall. Each exclusion shelter fixed on the stainless-steel beams was V-shaped and transparent to allow UV radiation through. To allow the excluded flow to drain from the plots, the slope of the shelter was adjusted to match the slope of the plots and connected to PVC troughs on the lower slope. Depending on the distribution of trees in the plots, each shelter had a width that varied from 0.7 to 1 m and covered 50% of the entire plot space. This study used a similar throughfall reduction rate (50%) to that used in other TR experiments in subtropical plantations due to the lack of lack of quantitative forecasts on the reduction of future rainfall in the area. The control plots were established without TR, but to stop the lateral water flow, the four edges of each control plot were trenched to 60–80 cm depth using PVC boards of 1 m height. In addition, to reduce the difference between control plots and TR plots, the litter on the exclusion shelters was regularly returned to the TR plots.

### Soil sampling and soil physicochemical properties analysis

On January 26 and July 29, 2021, five sampling points were selected randomly within each plot, collecting 0–20 cm and 20–40 cm soil samples. The soil samples were sieved through a 2-mm mesh immediately in the field after being cleared of litter, stones, and other dead soil animals. The five soil cores from the same plot were then combined to create a composite sample. Each composite sample was further divided into two subsamples, which were stored for later analysis of soil chemical properties (air-dried and stored at 4°C) and microbial communities (stored at −80°C). A total of 12 samples (2 precipitation treatments × 2 soil depths × 3 replicate plots) were obtained.

To determine soil water content (SWC), 10 g of fresh soil samples were oven dried at 105°C for 24 h and sampled three times a month. Soil pH was determined using a pH-meter (Mettler-Toledo GmbH, Greifensee, Switzerland) in a 1:2.5 soil/water suspension. Total organic carbon (TOC) was determined by the K_2_Cr_2_O_7_ titration method (Zhao et al., 2021). Total nitrogen (TN) was measured by the indophenol blue colorimetric method (Fang et al., 2008). Total phosphorus (TP) was extracted by H_2_SO_4_ digestion, available phosphorus (AP) was extracted using HClO_4_-H_2_SO_4_, and both were measured by the ascorbic acid/molybdate reagent blue color method (Zhao et al., 2021). Soil organic matter (SOM) was determined by the potassium dichromate oxidation external heating method (Tong et al., 2021). Alkali-hydrolyzed nitrogen (AHN) was determined by the alkali hydrolysis diffusion method (Qi et al., 2020). Total potassium (TK) was melted with sodium hydroxide and measured by flame photometry (Pan X. M. et al., 2020). Soil properties data in the dry season has been published in Lin et al., 2022.

### DNA extraction and PCR amplification

Total community DNA was extracted from *Eucalyptus* soil samples using an E.Z.N.A.® soil DNA Kit (Omega Bio-Tek, Norcross, GA, United States). The primers 338F (5′-ACTCCTACGGGAGGCAGCAG-3′) and 806R (5′-GGACTACHVGGGTWTCTAAT-3′) were used for amplifying the V3-V4 hypervariable regions of the bacterial 16S rRNA gene (Wear et al., 2018). For fungi, the ITS2 region was amplified using the primer pair ITS1F (5’-CTTGGTCATTTAGAGGAAGTAA-3′) and ITS2R (5’-GCTGCGTTCTTCATCGATGC-3′) (Lai et al., 2007). The PCR conditions for the 16S rRNA gene were aninitial denaturation at 95°C for 3 min, followed by 27 cycles of 95°C for 30 s, 55°C for 30 s, and 68°C for 45 s, and then a final extension at 72°C for 10 min. The PCR conditions for the ITS1 region were an initial denaturation at 95°C for 3 min, followed by 35 cycles of 30 s at 95°C, 55°C for 30 s, and 45 s at 72°C; and then a final extension at 72°C for 5 min.

### Library preparation and Illumina sequencing

PCR products were recovered using 2% agarose gel and purified using the AxyPrep DNA Gel Extraction Kit (Axygen Biosciences, Union City, CA, United States), then were eluted by Tris–HCl, and detected by 2% agarose electrophoresis using Quantifluor-ST (Promega, United States). The products were quantified according to Illumina MiSeq platform (Illumina, San Diego, United States) Standard Operating Procedures for generating sequencing libraries from the purified amplified fragments. Finally, purified amplicons were pooled in equimolar amounts and paired-end sequenced (2 × 300 bp) on an Illumina MiSeq platform according to the standard protocols.

### Bioinformatics analysis

This bioinformatics analysis was performed using the “Atacama soil microbiome tutorial” in the QIIME 2 documentation.^1^ Using the QIIME Tools import program, raw data FASTQ files were converted into a format that could be processed by the QIIME 2 system. We quality filtered and trimmed demultiplexed sequences from each sample, denoised, merged, and then identified and removed chimeric sequences using the DADA2 plugin for QIIME2 to obtain the feature table of amplicon sequence variants (ASVs) (Callahan et al., 2016). Subsequently, bacterial species were annotated based on the silva132 database^2^ and fungal species were annotated based on the UNITE 7.0 database^3^ to obtain the taxonomy table (confidence threshold is 70%). Any contaminating mitochondrial and chloroplast sequences were filtered using the QIIME 2 feature-table plugin. The raw reads of the dry and rainy season were deposited in the National Center for Biotechnology Information (NCBI) SRA database (accession numbers PRJNA774083 and PRJNA774151 for dry season, and PRJNA821707 and PRJNA821932 for rainy season). The raw reads of the dry season have been used in our previous study (Lin et al., 2022).

### Functional analysis

Phylogenetic Investigation of Communities by Reconstruction of Unobserved States (PICRUSt2) software was used for functional prediction of 16S rRNA gene data (Douglas et al., 2020), and the Kyoto Encyclopedia of Genes and Genomes (KEGG) database was utilized to predict KEGG Orthology (KO) function and KEGG metabolic pathway (KEGG pathway). An analysis of fungal functional classification was carried out using FUNGuild (Nguyen et al., 2016). Comparing fungal functional groups with “probable” and “high probability” was done based on confidence rankings in order to avoid overinterpreting. After that, a statistical analysis of the relative abundance of various functional groups was conducted.

### Statistical analyzes

The effects of season, TR treatment, and their interactions on soil physicochemical properties were analyzed using Generalized Linear Models (GLM) and the Kruskal-Wallis test. The differences in block design were considered and block design was added as a covariate to the GLM analysis. Alpha-diversity indices (Chao1, Shannon, Simpson and Pielou’s evenness)were calculated using QIIME 2 software.^4^ Non-metric multidimensional scaling (NMDS) analysis based on the Bray-Curtis distance was completed using the Wekemo Bioincloud^5^ to detect the difference between microbial community samples. Redundancy analysis (RDA) was performed using the Wekemo Bioincloud (see footnote 5) to identify the relationships between microbial abundance and soil chemical properties. Transform the datd with hellinger before performing RDA analysis. Linear discriminant analysis Effect Size (LEfSe) was performed using the Galaxy/Hutlab tool^6^ in order to determine differentiating features at species level. Results were displayed as histograms and cladograms, representing taxa with an LDA > 2.0 threshold (Segata et al., 2011). The statistical analysis of metagenomic profiles (STAMP) software was used to determine the functional abundance of bacterial differences between the soil samples, using two-sided Whites’s *t*-test, with Benjamini–Hochberg false discovery rate (FDR) correction for multiple testing. When comparing the differences between seasons, CK and TR were combined at both depths as dry season and rainy season. The structural equation models (SEM) for exploring the correlation among season, TR, soil physicochemical properties, microbial diversity, and microbial composition. Firstly, we selected the top three soil physicochemical properties in 0–20 cm and 20–40 cm soil depths through principal component analysis, then replaced the diversity and composition with Shannon index and most dominant phylum respectively, and finally constructed and modified by AMOS 26.0 software.

### Network analyzes

Network analyzes were performed to assess microbiota complexity. The top 500 dominant bacterial ASVs and top 100 dominant fungal ASVs in both soil depths were selected in order to prevent false correlations. These AVSs accounted for the top 66.26 and 97.28% of the relative abundance of bacteria and fungi in the dry season and the top 70.34 and 86.37% of the relative abundance of bacteria and fungi in the rainy season, respectively. Spearman’s rank correlation was used to assess the association among microbial ASVs from all time points. Corrections for multiple comparisons were performed for Spearman’s rank correlations using the FDR correction. The *fdrtool* package was used to calculate correlation networks in R. Only correlation coefficients where *r* > 0.7 or *r* < −0.7 (significant at *p* < 0.05) are shown. Gephi software was used to visualize the network structure (Bastian et al., 2009) based on the undirected network (directionless edges) and the Fruchterman–Reingold layout.

## Results

### Soil physicochemical properties

Ten physicochemical properties of soil were measured (Supplementary Figure S1). In both soil depths (0–20 and 20–40 cm), the seasonal variations had a stronger effect on soil nutrients compared with the TR treatment. In the 0–20 cm soil depth, TN, TP, AHN, TOC, pH, C/N ratio, and pH were higher in the dry season than in the rainy season. Moreover, the interaction of TR treatment and season had a significant effect on AP and AHN at 0–20 cm soil depth. TR treatment increased TOC, AHN, and pH. In the 20–40 cm soil depth, TP, AHN, TOC, pH, and C/N ratio were also significantly higher in the dry season than in the rainy season. Compared with control (CK) treatment, TR significantly reduced soil water content (SWC) at 0–20 cm and 20–40 cm soil depths by 5.04 and 5.29% (from July 2020 to September 2021), respectively. In addition, TR significantly reduced SWC in the rainy season but not in the dry season and only at 0–20 cm depth (Figure 2).

### Diversity and compositions of soil microbial communities

A total of 679,766 high-quality bacterial sequences (from 45,329 to 71,211 per sample) and 754,724 high-quality fungal sequences (from 53,170 to 74,021 per sample) were obtained from dry season soil samples. In addition, a total of 682,260 high-quality bacterial sequences (from 38,453 to 65,463 per sample) and 945,675 high-quality fungal sequences (from 69,399 to 92,282 per sample) were obtained from rainy season soil samples. The species richness in samples and the rationality of the sequencing data may both be seen in the rarefaction curve. When the curve tends to be flat, the amount of sequencing data meets the test requirements and can characterize the microbial diversity in soil samples. The number of Observed_ASVs increased with the increase in the number of sequences, and the curve showed a trend of gradual increase and then flattening, indicating that the sequencing data were reasonable, and the sequencing depth could reflect the species information in the samples (Supplementary Figure S2).

**Figure 3:**
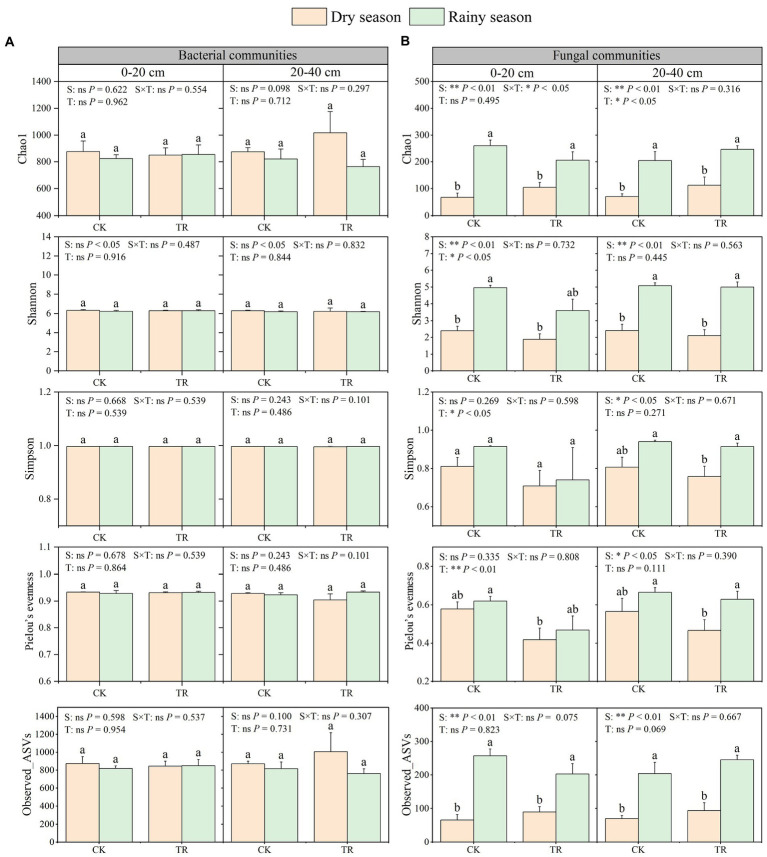
Alpha-diversity indices of bacterial **(A)** and fungal **(B)** communities in *Eucalyptus* plantations. Data are expressed as means + standard error (*n* = 3). Statistically significant differences (*p* < 0.05) between treatments are indicated by lowercase letters based on the Kruskal-Wallis test. CK, control; TR, throughfall reduction treatment. Data from GLM analyzes are indicated at the top of each graph; S, season, including dry season and rainy season; T, treatment, including TR and CK; S × T, the interaction of season and treatment; ‘*’, ‘**’, and ‘ns’ refer to *p* < 0.05, *p* < 0.01, and *p* > 0.05, respectively. Block design was added to the GLM analysis as a covariate.

Alpha-diversity analyzes showed that season and TR treatment had little effect on soil bacterial diversity (Figure 3A). On the contrary, season variation markedly influenced soil fungal community diversity (Figure 3B). In the 0–20 cm soil depth, Chao1,Shannon, and Observed_ASVs indices of soil fungi were higher in the rainy season than in the dry season. In addition, Shannon and Simpson indices and Pielou’s evenness of soil fungi were both significantly lowered by TR treatment. In the 20–40 cm soil depth, the Chao1, Shannon, Simpson, Pielou’s evenness, and Observed_ASVs indicesof soil fungi were higher in the rainy season ompared with those in the dry season (Figure 3B).

**Figure 4:**
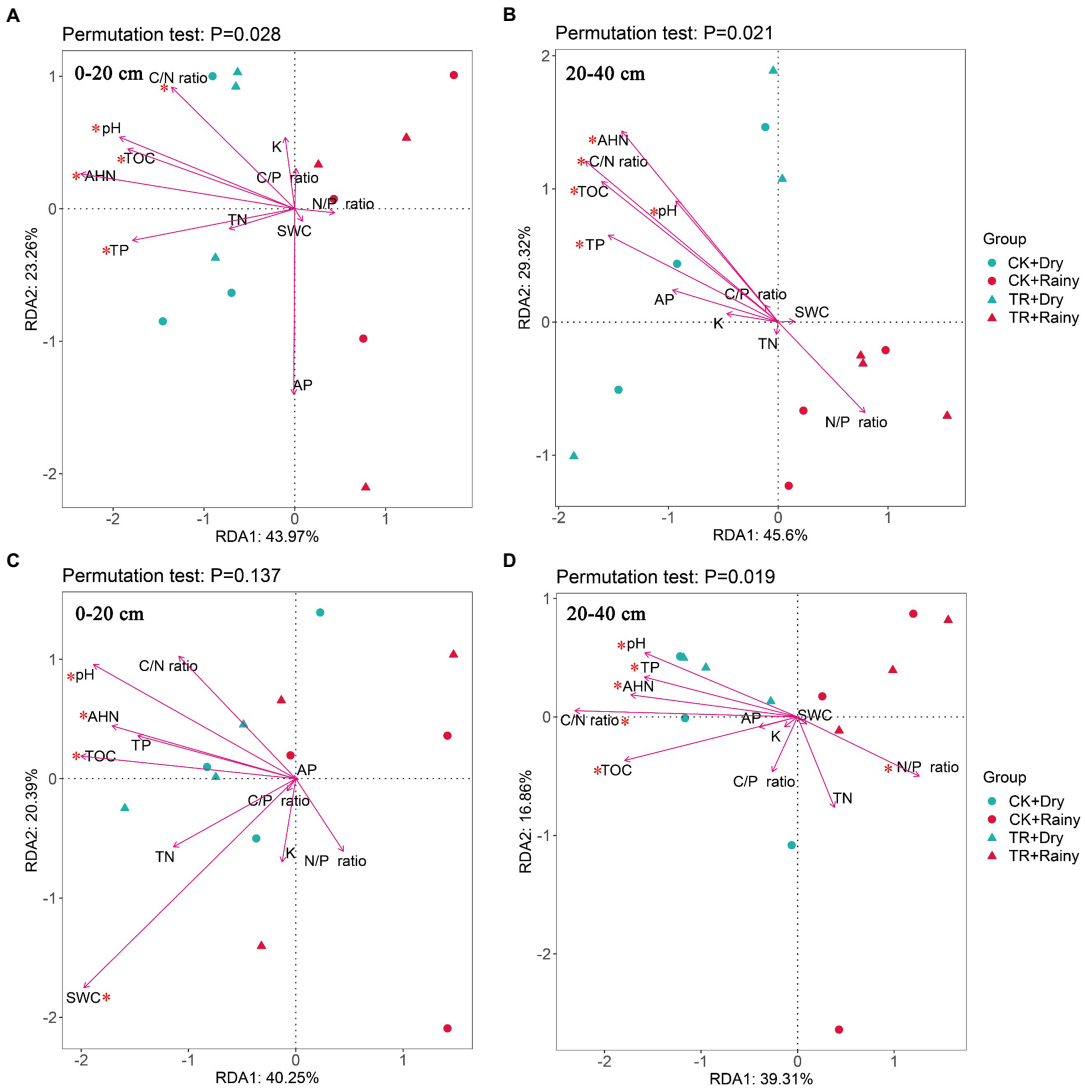
Redundancy analysis (RDA) illustrating the relationships between bacterial **(A,B)** or fungal **(C,D)** communities and soil physicochemical properties. TN, total nitrogen. TP, total phosphorus; TK, total potassium; TOC, total organic carbon; AP, available phosphorus; SWC, soil water content; AHN, alkali hydrolyzed nitrogen; C/N ratio, the ratio of total organic carbon and total nitrogen; C/P ratio, the ratio of total organic carbon and total phosphorus; N/P ratio, the ratio of total nitrogen and total phosphorus. *p*-values represent the result of PERMANOVA testing with 999 permutations.

The NMDS analysis revealed that there were seasonal separations in the fungal community at soil depths of 0–20 cm and 20–40 cm (Supplementary Figures 3C,D). Dry CK and TR are grouping together and separately from rainy CK and TR. For RDA results based on phylum abundance data, the bacterial communities at both soil depths were correlated with TP, AHN, TOC, pH, and C/N ratio (Figures 4A,B; Supplementary Table S1). The relative abundances of Proteobacteria and Actinobacteria were positively related to TP, AHN, TOC, pH, and C/N ratio, while the relative abundances of Acidobacteria and Chloroflexi were negatively related with these soil physicochemical properties. Fungal communities at 20–40 cm soil depth were significantly correlated with TP, AHN, TOC, pH, C/N ratio, and N/P ratio (Figure 4D; Supplementary Table S2). The relative abundance of Basidiomycota was positively correlated with TP, AHN, TOC, pH, C/N ratio, and N/P ratio. However, these soil physicochemical qualities were inversely correlated with the relative abundance of Ascomycota.

**Figure 5:**
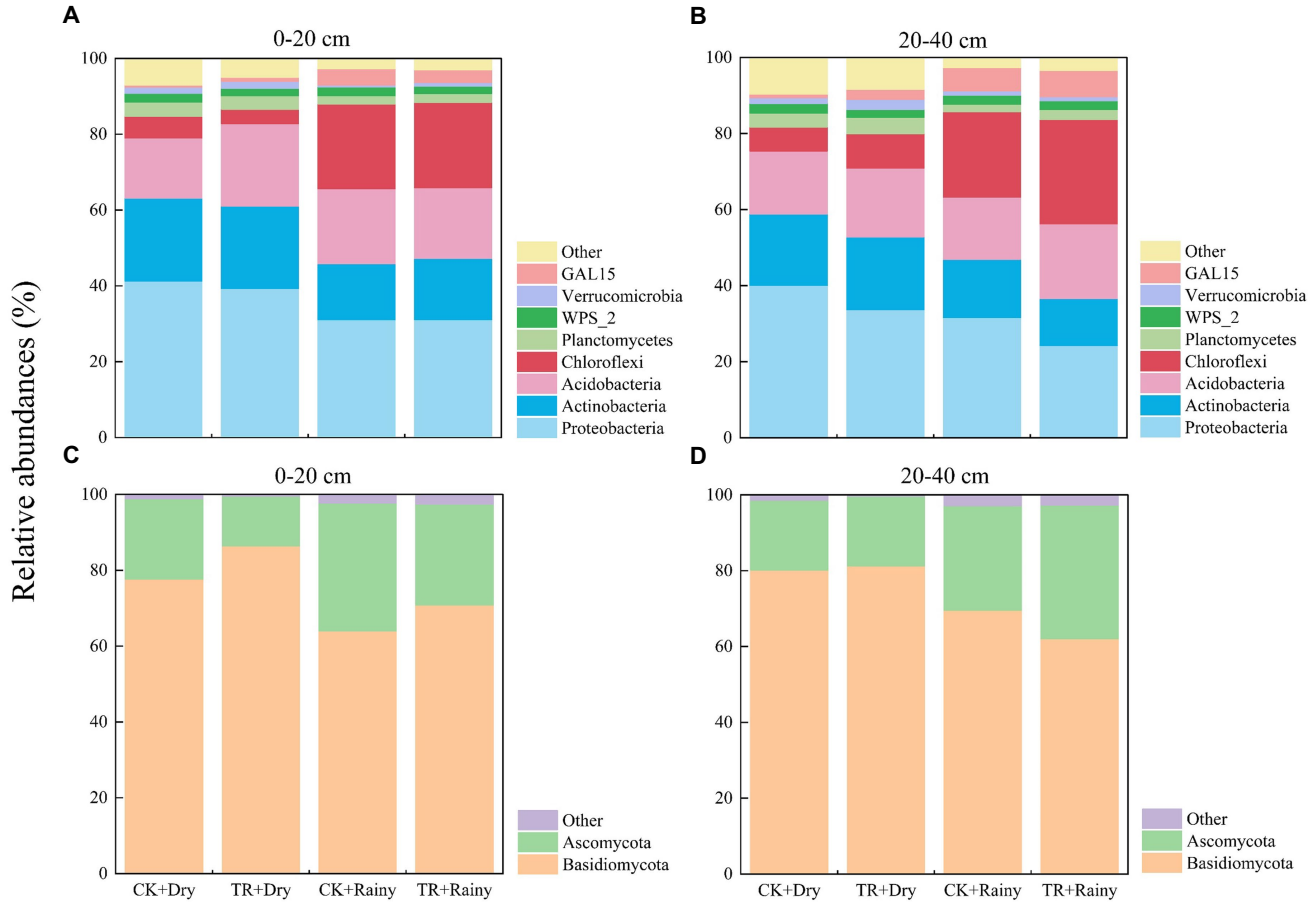
Relative abundances of the dominant bacterial **(A,B)** and fungal **(C,D)** phyla under different treatments. CK, control; TR, throughfall reduction treatment; Dry, the dry season; Rainy, the rainy season. Phyla accounting for ≥1% are shown while those <1% and unclassified group are integrated into ‘other’.

At the phylum level, dominant bacteria and fungi were those having defined as those having a relative abundance of more than 1%. The dominant bacterial phyla in *Eucalyptus* soil were Proteobacteria (relative abundance: 24.28–41.28%), Actinobacteria (12.29–21.82%), Acidobacteria (15.84–21.73%), Chloroflexi (5.75–27.40%), Planctomycetes (2.04–4.24%), GAL15 (0.58–6.92%), WPS_2 (1.86–2.55%), and Verrucomicrobia (0.49–2.65%; Figures 5A,B). The dominant fungal phyla were Basidiomycota (61.98–86.38%) and Ascomycota (13.19–35.28%; Figures 5C,D).

LEfSe showed that *Eucalyptus* soil microorganisms had significantly different community compositions in the dry and rainy seasons. For the bacterial community, the bacterial taxa responsible for the different bacterial community structures between the dry and rainy seasons were different. More bacterial taxa contributed significantly to the bacterial community structure in the dry season compared with the rainy season, these taxa included TK17, Rhodospirillaceae, *Nitrosovibrio*, etc., whereas only Unclassified_Rhizobiales and *Crossiella* were present in the rainy season (Supplementary Figure S4). Unlike the bacterial community, very few fungal taxa play a role in seasonal variation in fungal community composition. The fungal taxa that caused differences in the structure of the fungal community in the dry season were Tricholomataceae and Unclassified_Eurotiales, while those that caused differences in the structure of the fungal community in rainy season were Trichomeriaceae, *Hyphodontia*, Xylariaceae, Schizoporaceae, and Lecanoromycetes (Supplementary Figure S5).

### Co-occurrence networks analyzes

**Figure 6:**
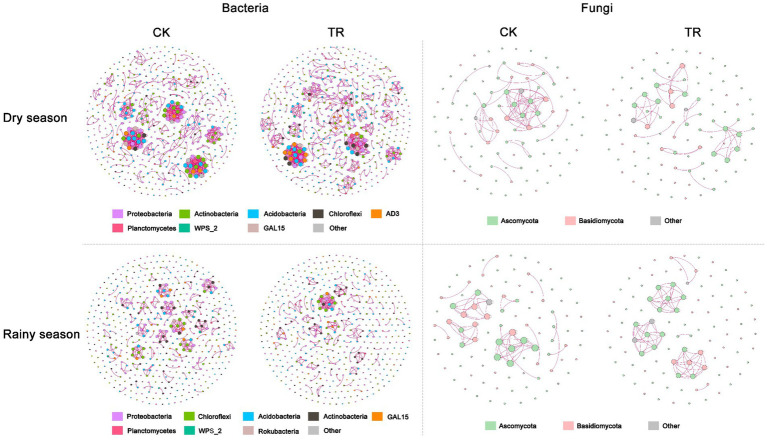
Bacterial and fungal networks under CK and TR treatment in dry and rainy seasons. Both soil depths were used to perform these co-occurrence network analyze. Only Spearman’s correlation coefficients where r > 0.7 or r < −0.7 (significant at *p* < 0.05) are shown. The nodes are colored according to phyla. Positive correlations are indicated by red borders, whereas negative correlations are indicated by blue edges. Node size is proportional to the betweenness centrality of each ASV, and edge thickness is proportional to the weight of each correlation.

Network analysis showed that the community structure of soil bacterial differed between the dry and rainy seasons (Figure 6). Compared with the dry season, the bacterial community in the rainy season had a less complex network with fewer edges (855 and 785), a lower average degree (3.42 and 3.14), and a lower average path length and diameter (Supplementary Table S3). In contrast, fungal networks were similar in the dry and rainy seasons and were less complex compared with the bacterial networks (Figure 6). Moreover, TR treatment had no discernible impact on the network characteristics of the bacterial and fungal communities compared with the control (CK) (Figure 6; Supplementary Table S3).

### Response of functional groups of bacteria and fungi to throughfall reduction

**Figure 7:**
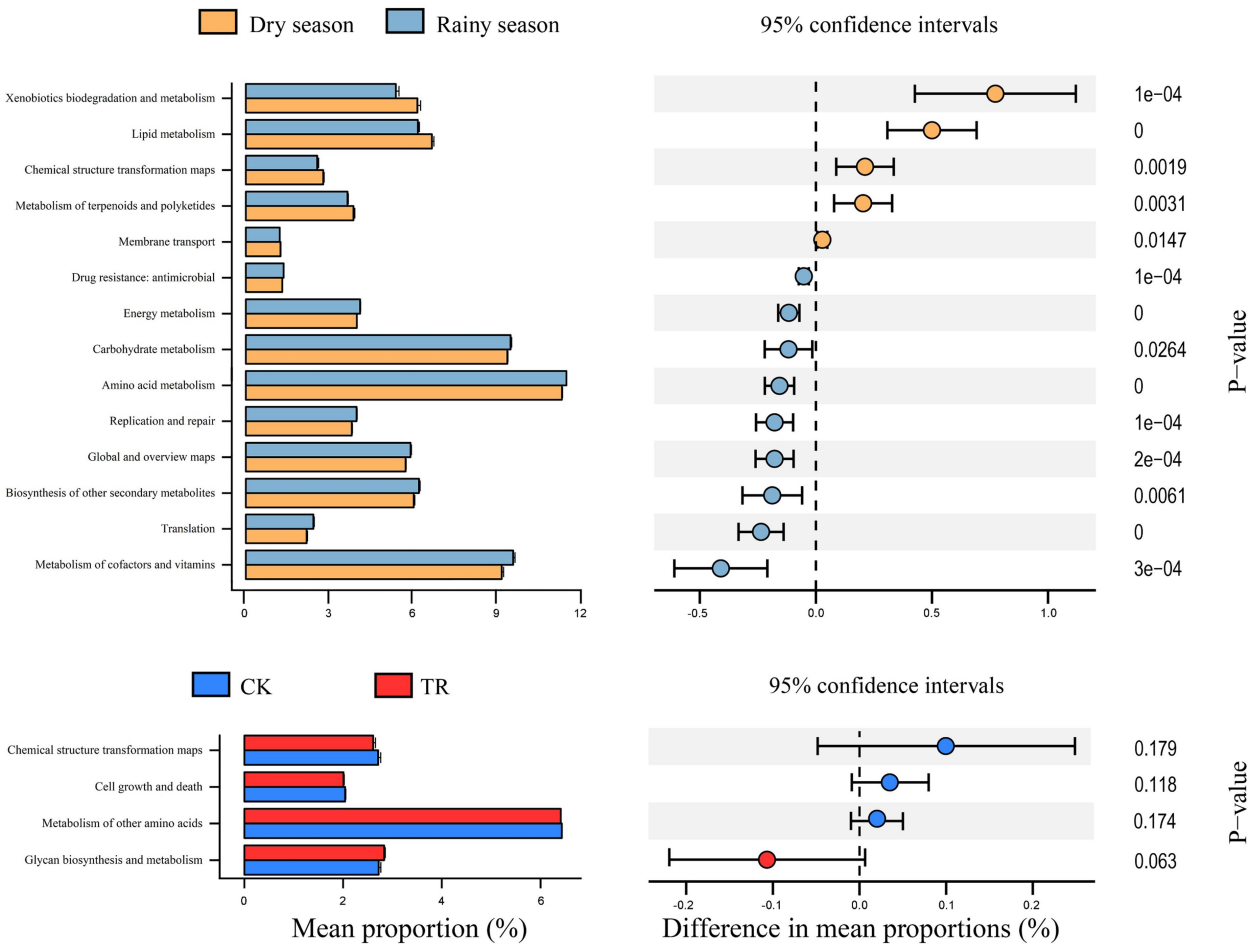
STAMP analysis of bacterial function prediction (hierarchy level 2). In the figure, Y-axis is the pathway predicted by function. Column length is the average relative abundance of the pathway in each sample group. CK: control; TR: throughfall reduction treatment.

Based on PICRUSt2 and the KEGG database, functional analysis of the bacterial community was performed to obtain functional prediction information. Six biometabolic pathways were present in all samples: metabolism, genetic information processing, etc. (Supplementary Figure S6). The metabolism pathway exhibited the highest relative abundance in all samples. STAMP analysis showed that the relative abundances of metabolism, human diseases, organismal systems, and environmental information processing in the dry season were significantly higher than those in the rainy season, and there were no significant differences between CK and TR (Supplementary Figure S6). Further analysis revealed a total of 47 secondary functions; the top 20 in terms of relative abundance were environmental adaptation, cell growth and death, and carbohydrate metabolism, etc. (Supplementary Figure S7). Figure 7 illustrates that seasonal differences have a greater impact on the relative abundance of secondary function depth genes compared with TR treatment.

**Figure 8:**
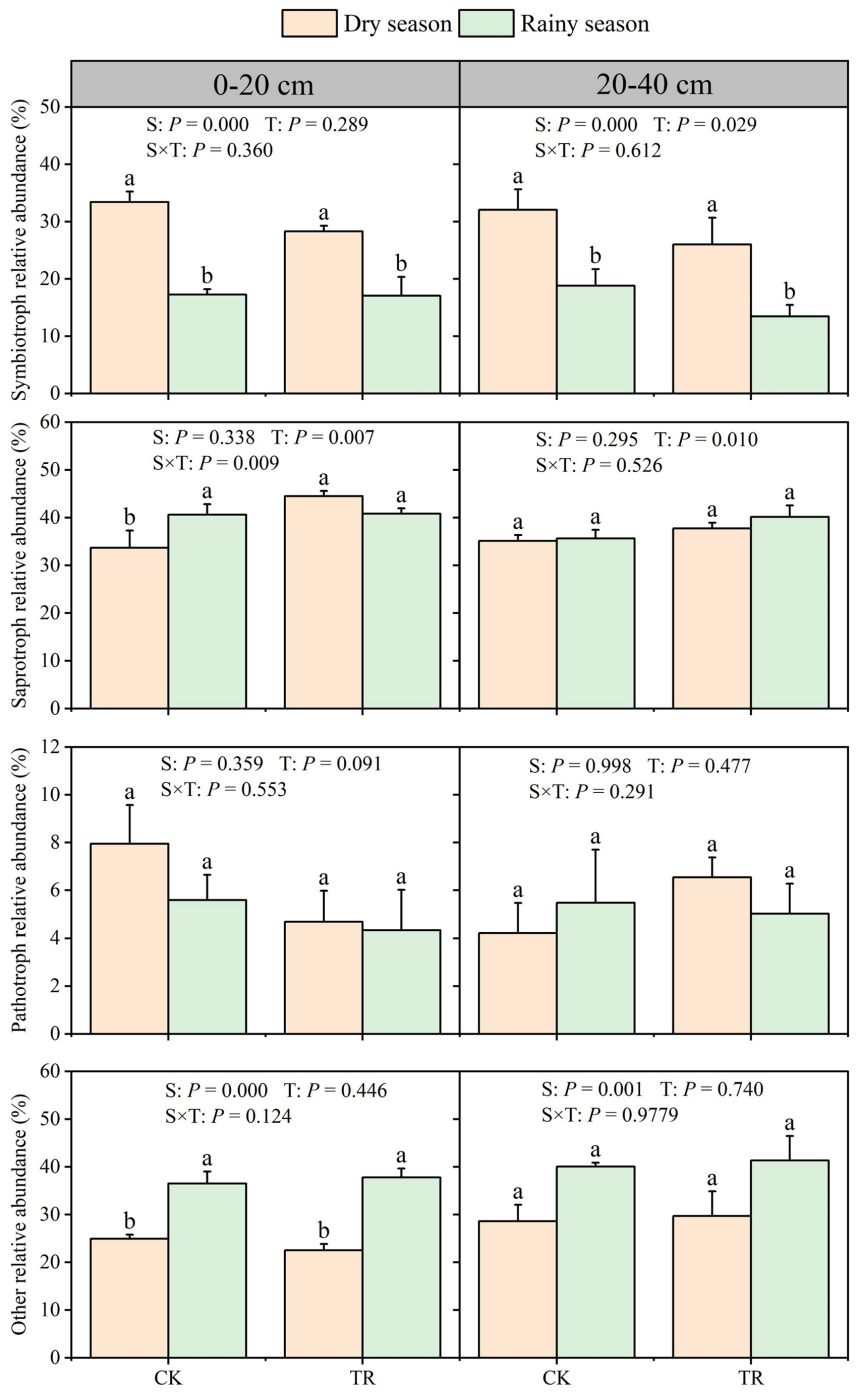
Nutritional mode of soil fungal community. Data are expressed as means + standard error (*n* = 3). Statistically significant differences (*p* < 0.05) between treatments are indicated by lowercase letters based on the Kruskal-Wallis test. CK: control; TR: throughfall reduction treatment. *p*-values from GLM analyzes are indicated at the top of each graph; S, season, including dry season and rainy season; T, treatment, including TR and CK; S × T, the interaction of season and treatment.

The fungal community of CK and TR treatment in the dry and rainy seasons were compared using the FUNGuild database. In the 0–20 cm soil depth, the dominant nutritional mode of fungi in the CK group in the dry season saprotroph (33.71%), followed by symbiotroph (33.43%), while other types and pathotroph accounted for 24.91 and 7.95%, respectively. Following TR treatment, saprotroph accounted for 44.53%, followed by symbiotroph (28.29%), other types (22.51%), and pathotroph (4.67%). In contrast, the relative abundance of symbiotrophic fungi significantly decreased in the rainy season (Figure 8). The same pattern was observed in the 20–40 cm soil depth, the relative abundance of symbiotrophs was significantly lower in the rainy season compared with that in the dry season (Figure 8).

### Structural equation model

**Figure 9:**
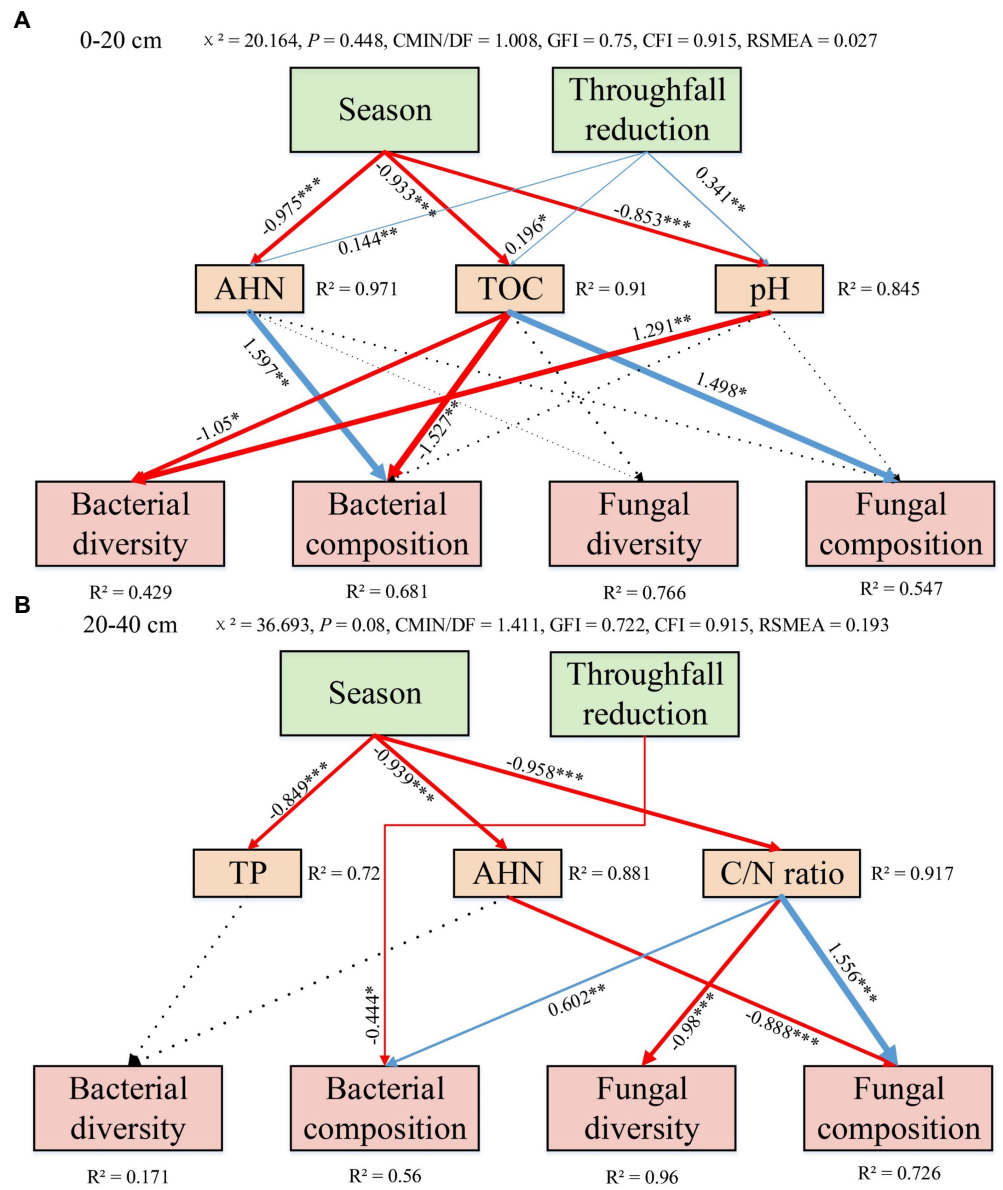
Structural equation model (SEM) of season variation and throughfall reduction effect on diversity and composition of soil bacterial and fungal communities at 0–20 cm **(A)** and 20–40 cm **(B)** soil depth. Blue arrows indicate positive effects, and red arrows indicate negative effects. The thickness of the line of the arrow indicates the correlation strength. The number next to the arrow represents the standardized path coefficient, indicating the correlation strength of the two factors. *, **, and *** indicate statistical significance levels at *p* < 0.05, *p* < 0.01 and *p* < 0.001, respectively. AHN, alkali hydrolyzed nitrogen; TOC, total organic carbon; TP, total phosphorus; C/N ratio, the ratio of total organic carbon and total nitrogen.

At 0–20 cm soil depth, the path coefficients of SEM showed that soil bacterial diversity was predominantly negatively influenced by TOC (−1.05) and pH (−1.291) (Figure 9A); soil bacterial composition was predominantly positively influenced by AHN (1.597) and negatively influenced by TOC (−1.527) (Figure 9A); and soil fungal composition was mainly positively influenced by TOC (1.498) (Figure 9A). At 20–40 cm soil depth, different parameters influenced bacterial and fungal diversity and composition (Figure 9B). The path coefficients of SEM showed that soil bacterial composition was predominantly negatively influenced by TR (−0.444) and positively influenced by C/N ratio (0.602) (Figure 9B); soil fungal diversity was mainly negatively influenced by C/N ratio (−0.98); and soil fungal composition was mainly predominantly influenced by AHN (−0.888) and C/N ratio (−1.556) (Figure 9B).

## Discussion

In this study, the effects of season on the composition and diversity of bacterial and fungal communities in *Eucalyptus* soil were used Illumina high-throughput sequencing of 16S rRNA and ITS genes. Microbial biodiversity and soil physicochemical properties affect soil degradation or amelioration processes (Nie et al., 2018). Furthermore, the activity and growth of plants and soil microbes are affected by water availability (Engelhardt et al., 2021). Congruent with previous reports (Zhao et al., 2017; Su et al., 2020; Lin et al., 2022), this study showed that TR significantly reduced SWC in the rainy season. For other soil physicochemical properties, their contents were almost all reduced in the rainy season relative to those in the dry season. These findings are consistent with the results of Xue et al. (2003), which showed that the content of soil AP, TN, and TP in a *Parashorea chinensis* forest decreased during the rainy season. For some physicochemical properties, there are differences between TR and CK in the same season (AHN, for exaplme), indicating that soil physicochemical properties are predominantly influenced by seasonal variations. The rlower content of soil physicochemical properties in the rainy season compared with the dry season maight be due to *Eucalyptus* hybrids haveing a long fast-growth stage (>10 years); onsequently, the period of abundant rainfall is more conducive to the rapid growth of *Eucalyptus*, and thus there is a requirement for more soil nutrients with the development of forest stands (Zhou et al., 2017). It is also possible that the strong leaching effect of precipitation on the soil lead to large runoff of elements and nutrients in the soil with water.

For soil microorganisms, various biological and abiotic factors [such as vegetation (Weinert et al., 2011), soil properties (Wu et al., 2008), and soil texture (Schutter et al., 2001)] can affect their diversity and community composition. Our research found that seasonality is a powerfull driver force of soil fungal diversity and composition in a subtropical *Eucalyptus* plantation. In this study, Chao1, Shannon, Simpson, Pielou’s evenness and Observed_ASVs indices of soil fungal communities were all increased in the rainy season (Figure 3). Specifically, an increase in precipitation led to an increase in fungal diversity. Similar results were observed from a rubber plantation (Wei et al., 2022). Regarding soil bacteria, the relative abundance of the Chloroflexi decreased during the rainy season. This observation was contradicted the findings of Maestre et al. (2015), which showed that the abundance of Chloroflexi increased linearly with the increase of drought. Possible reasons for these discrepant results need to be further explored. In terms of soil fungal abundance, Basidiomycota and Ascomycota were the dominant fungi in *Eucalyptus* plantations. Moreover, there was a tendency for Ascomycota to be more numerous and Basidiomycota to be less numerous during the wet season. A previous study concluded that the abundance of Ascomycota increased with the onset of rainy seasons in rainforests (Brockett et al., 2012). It has been reported that in the forests of northern China, the abundance of Ascomycota was directly proportional to SWC, while the abundance of Basidiomycota is inversely proportional to SWC (He et al., 2016). Thus, it can be considered that Ascomycota and Basidiomycota exhibit opposite reactions to SWC. Furthermore, this demonstrates that these two dominant groups compete with one another for soil moisture (Lin et al., 2022). From the soil physicochemical properties (Supplementary Figure S1), microbial alpha-diversity (Figure 3), and co-occurrence networks analysis (Figure 6; Supplementary Table S3), it can be concluded that seasonal variations were a stronger driver than TR treatment. This partly proves our second hypothesis that seasonal variations in soil fungal communities had a more significant effect compared with TR treatment. Soil moisture and soil temperature both change with the seasons, and both play an important role in influencing soil microbes (Tabuchi et al., 2008). It has been suggested that differences in SWC lead to changes in bacterial community composition (Rasche et al., 2011). In contrast, another study found that changes in SWC did not markedly impact bacterial community structure (Stres et al., 2008). Our results showed that SWC had minimal effect on bacterial community composition or diversity (Figures 3, 4; Supplementary Figure S3). The RDA results demonstrated that the most important soil factors causing the change of bacterial community structure were AHN (0–20 cm depth) and C/N ratio (20–40 cm depth; Supplementary Table S1), while those causing the change of fungal community structure were SWC (0–20 cm depth) and TOC (20–40 cm depth; Supplementary Table S2). The SEM revealed that season influences the physicochemical parameters, and these ones influence the microbial diversity and composition. On the contrary, TR treatment is the one that directly influences bacterial composition. Co-occurrence network analysis showed that seasonal variations have a greater impact on the soil bacterial networks than on the fungal networks, and the structure of the soil bacterial networks in the dry season is more complex compared with those in the rainy season. Simultaneously, compared with fungal networks, bacterial co-occurrence networks are more complex. This confirms our third hypothesis. It has been shown that drought increases network connectivity and centrality in soil bacterial networks. It’s possible that drought affects bacterial co-occurrence networks over a lengthy period of time by altering the vegetation and lowering soil humidity.

Based on PICRUSt2’s prediction of bacterial community function in TR and CK samples, all samples contain six different kinds of biological metabolic pathways, together with 46 sub-functions. Among them, five functions (hierarchy level 1) and 14 functions (hierarchy level 2) were significantly affected by seasonal variations (Figure 7), and the majority of functional genes were all related to metabolism demonstrating that metabolism is instrumental in the *Eucalyptus* forest soil. Soil metabolism is generally based on the uptake of carbohydrates, amino acids, and energy to sustain bacterial survival, thus becoming the most dominant functional gene in the bacterial community. In addition, among the sub-functions highlighted in this study, the one with the highest abundance was amino acid metabolism, which was significantly higher in the rainy season than in the dry season. It may be that sufficient water increases the activity of microorganisms, resulting in enhanced amino acid metabolism. Fungi are classified into three main functional groups based on how they obtain nutrients: pathotrophs, saprotrophs, and symbiotrophs. Saprophytic and symbiotic fungi are essential for the cycling of nutrients in soil. Among them, saprophytic fungi decompose litter and produce organic matter, while symbiotic fungi provide nutrients that help plants to survive in poor living conditions. In this investigation, the number of symbiotic fungi was decreased significantly in the rainy season (Figure 8). One possible explanation for this is that the prolonged torrential rains and wet deposition of atmospheric nitrogen in the rainy season in southern China led to soil acidification (Lu et al., 2014). An earlier study discovered that soil acidification was the primary factor inhibiting the growth of arbuscular mycorrhiza (AM) fungi (Pan S. et al., 2020), the most widely distributed group of symbiotic fungi.

This study does have some limitations in experimental design and statistical analysis. First, owing sample plot size constraints, only three replicates for each group were included; in general, it is recommended to have at least five biological replicates in each group. Secondly, the soil temperature of the different groups was not measured. Research has showed that soil temperature regulates soil microbial community composition in a subtropical forest (Fang et al., 2016); however, there is also evidence that experimental TR does not affect soil temperature (Lu et al., 2017).

## Conclusion

Throughfall reduction significantly reduced soil water content in the rainy season, but had limited impacts on other soil physicochemical properties, which are predominantly influenced by seasonal variations. Fungal alpha-diversity decreased in the rainy season while bacterial alpha-diversity did not change significantly between dry and rainy seasons. In addition, seasonal variations affected bacterial networks but not fungal networks, suggesting that soil bacterial networks are more unstable compared with fungal networks under dry conditions. Functional prediction revealed that the expression of soil bacterial metabolic functions and symbiotic fungi decreased in the rainy season. In summary, seasonal variations have a stronger effect on soil bacterial and fungal community composition, diversity, and function compared with TR treatment.

## Data availability statement

The datasets presented in this study can be found in online repositories. The names of the repository/repositories and accession number(s) can be found in the article/Supplementary material.

## Author contributions

JL and QQ designed the study and provide financial support. YL, ZC, YG, JK, and LY participated in soil nutrient determination. YS, QH, and YL analyzed the data. YL was responsible for writing original draft and making figures, reviewed and edited by QQ. All authors contributed to the article and approved the submitted version.

## Funding

This study was funded by the Forestry Science and Technology Innovation Project of Guangdong Province (2022KJCX015), and the National Key Research and Development Program of China (2016YFD0600201 and 2016YFD060020102).

## Conflict of interest

The authors declare that the research was conducted in the absence of any commercial or financial relationships that could be construed as a potential conflict of interest.

## Publisher’s note

All claims expressed in this article are solely those of the authors and do not necessarily represent those of their affiliated organizations, or those of the publisher, the editors and the reviewers. Any product that may be evaluated in this article, or claim that may be made by its manufacturer, is not guaranteed or endorsed by the publisher.

## Footnotes

^1^ https://docs.qiime2.org/2019.1/

^2^ https://www.arb-silva.de/

^3^ https://unite.ut.ee/

^4^ https://qiime2.org/

^5^ https://www.bioincloud.tech

^6^ http://huttenhower.sph.harvard.edu/galaxy/
