# Supplementary material for: Seasonal variations of soil bacterial and fungal communities in a subtropical Eucalyptus plantation and their responses to throughfall reduction
Source: Front Microbiol. 2023 Mar 28;14:1113616. doi: 10.3389/fmicb.2023.1113616 (PMC10086269; doi:10.3389/fmicb.2023.1113616)
Supplement: Supplementary file 1 [file Data_Sheet_1.docx]

**Supplementary materials for**

**Seasonal variations of soil bacterial and fungal communities in a** **subtropical *Eucalyptus* plantation and their responses to throughfall reduction**

Yubiao Lin, Ling Yang, Zetao Chen, Yaqian Gao, Jiejun Kong, Qian He, Yan Su, Jiyue Li and Quan Qiu^*^

Guangdong Key Laboratory for Innovative Development and Utilization of Forest Plant Germplasm, College of Forestry and Landscape Architecture, South China Agricultural University, Guangzhou 510642, China;

^*^ Correspondence author. 483 Wushan Road, Guangzhou 510642, China.

E-mail address: qqiu@scau.edu.cn (Quan Qiu)

**Summary:** The supplementary materials contained 7 supplementary Figures and 3 supplementary Tables (Supplementary Figures 1-7, Supplementary Tables 1-3).


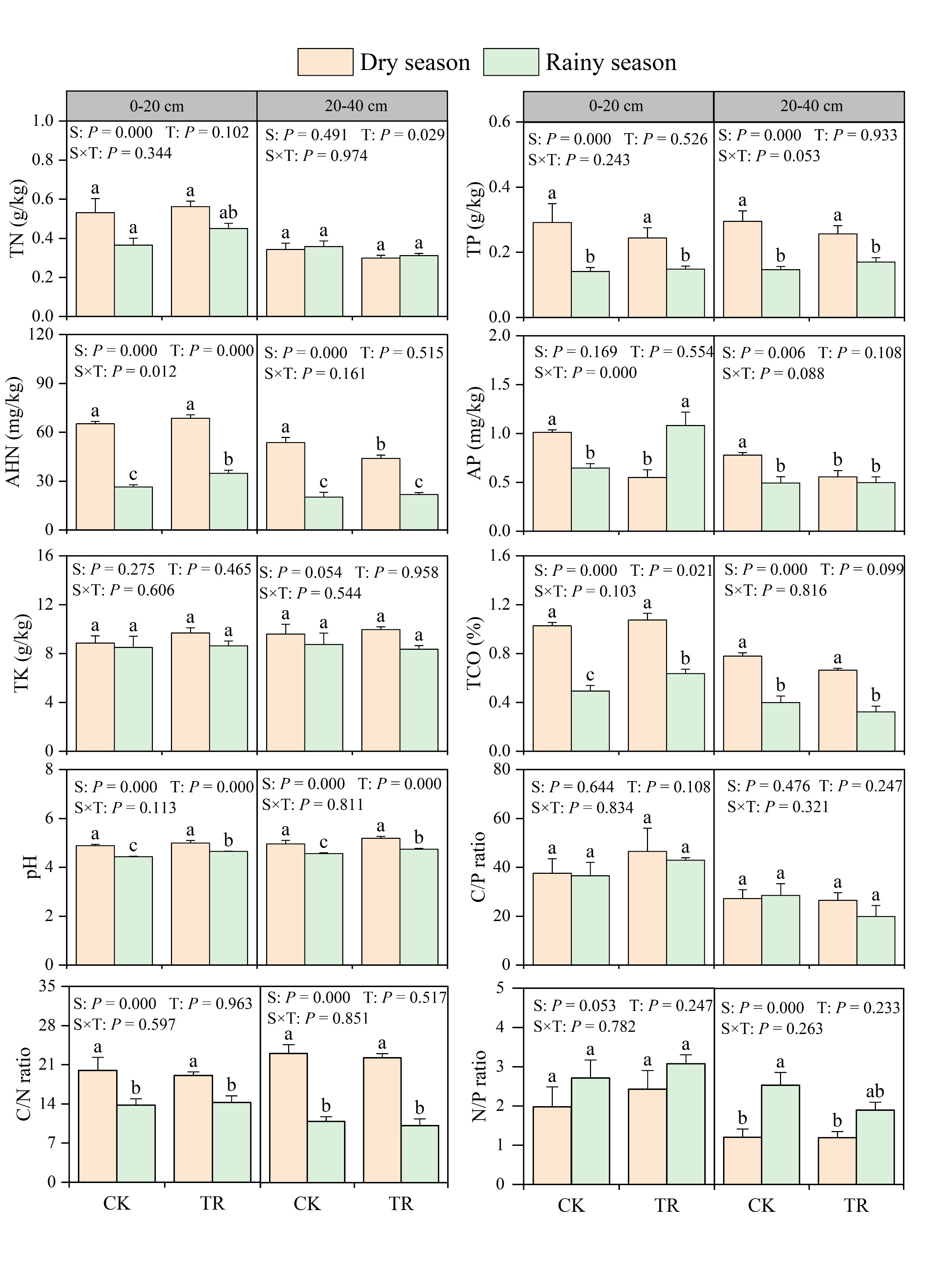


**Supplementary Figure 1.** Soil physicochemical properties. TN, total nitrogen. TP, total phosphorus. TK, total potassium. TOC, total organic carbon. AP, available phosphorus. AHN, alkali hydrolyzed nitrogen. C/N ratio, the ratio of total organic carbon and total nitrogen. C/P ratio, the ratio of total organic carbon and total phosphorus. N/P ratio, the ratio of total nitrogen and total phosphorus. Values are means + standard error (n = 3). Different lowercase letters indicate significant differences between treatments. CK: control; TR: throughfall reduction treatment. S, season, including dry season and rainy season; T, treatment, including TR and CK. S×T, the interaction of season and treatment. *P*-value represents the result of GLM analysis. Block design has been added to GLM analysis as a covariate.**
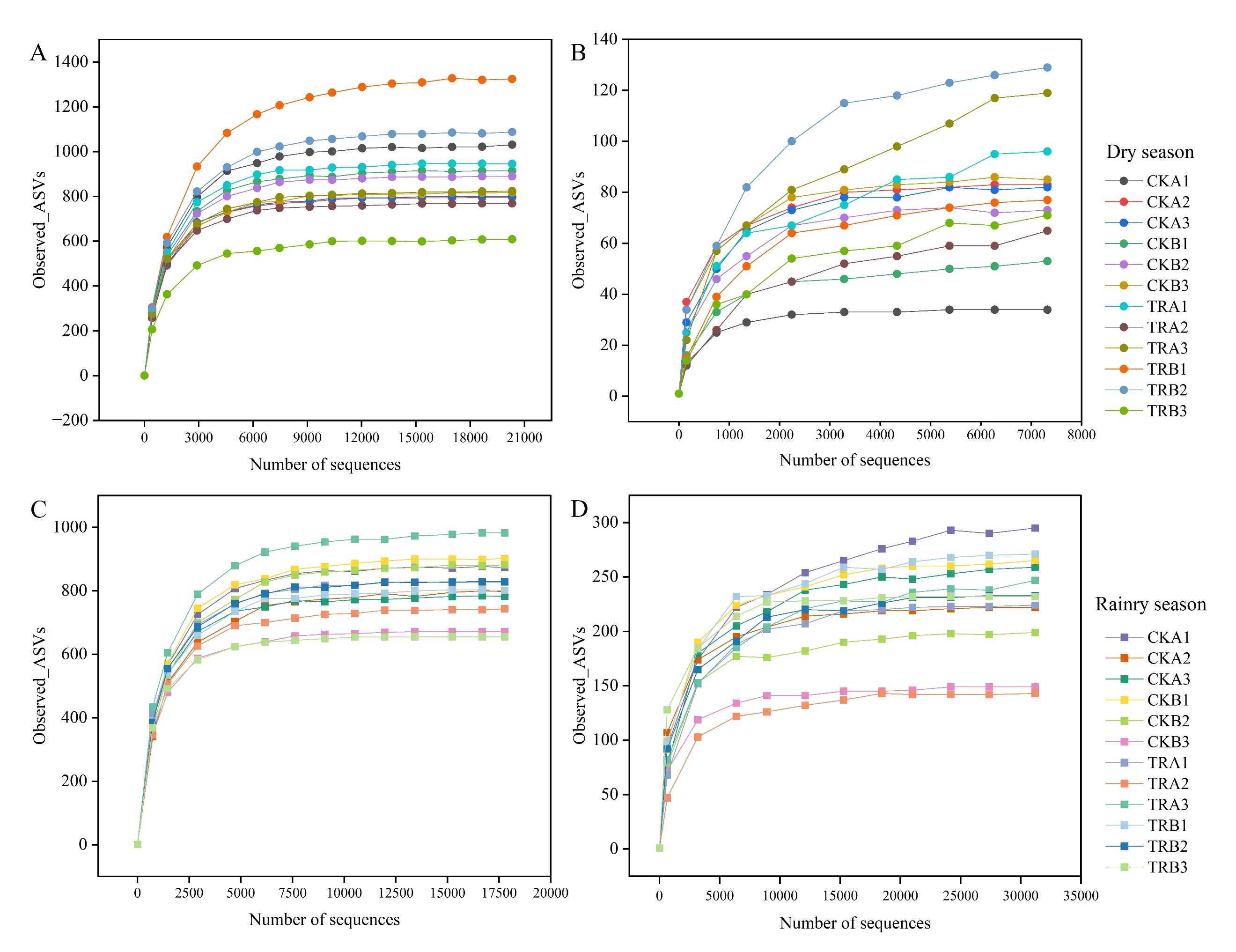
**

**Supplementary Figure 2.** Rarefaction curves of all sample are generated for bacterial (A, C) and fungi (B, D) AVSs. CKA, control with 0–20 cm soil depth; CKB, control with 20–40 cm soil depth; TRA: throughfall reduction with 0–20 cm soil depth; TRB, throughfall reduction with 20–40 cm soil depth. CKA1, CKA2, and CKA3 represent three replicates of CKA, and so on.


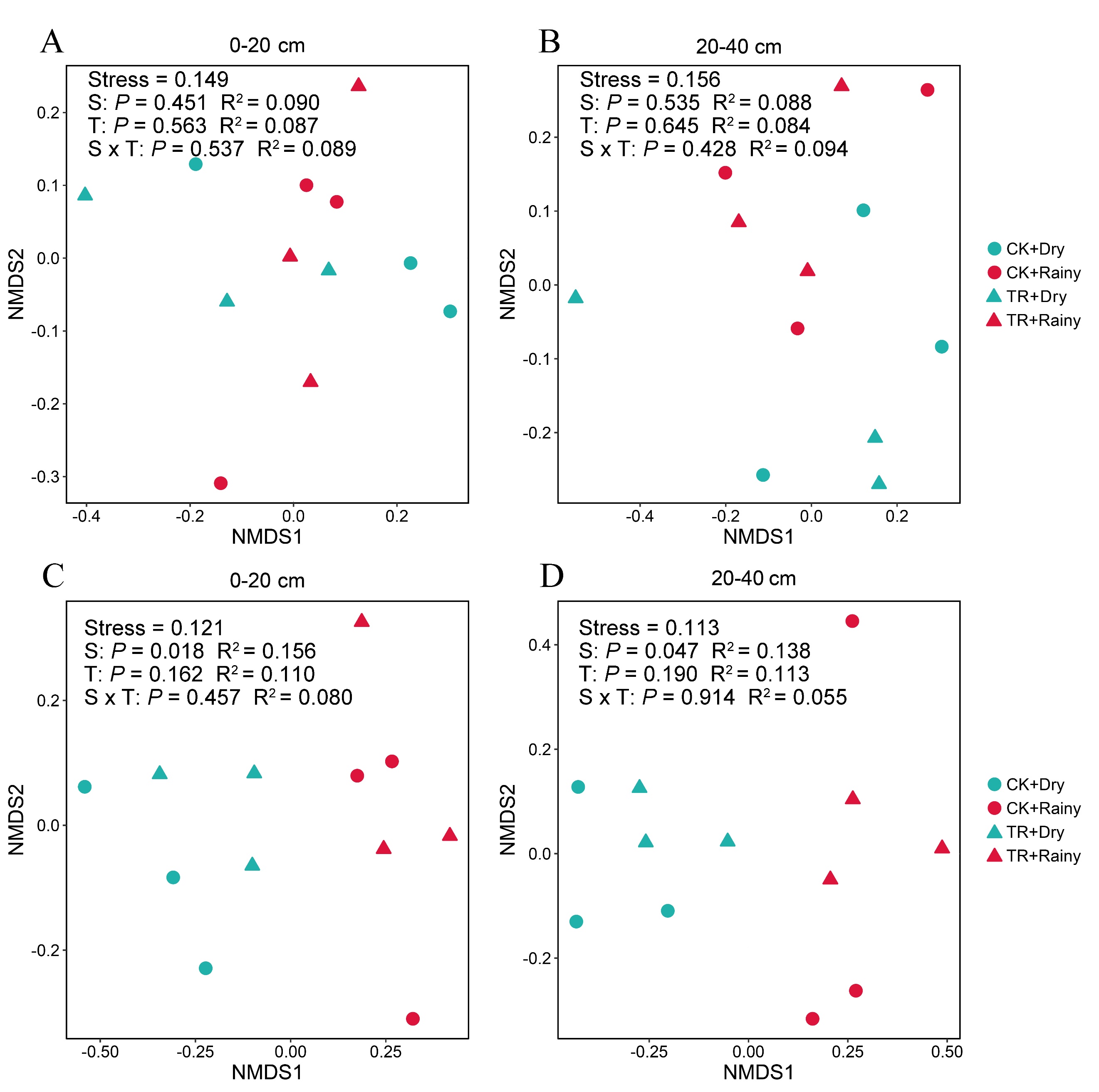


**Supplementary Figure 3.** Non-metric multidimensional scaling (NMDS) analysis based on the Bray-Curtis distance for the bacterial (A, B) and fungal (C, D) communities. CK: control; TR: throughfall reduction treatment. Dry: the dry season; Rainy: the rainy season. S, season, including dry season and rainy season; T, treatment, including TR and CK. S×T, the interaction of season and treatment. *P*-value and the explained variances values (R^2^) represent the result of PERMANOVA.

**
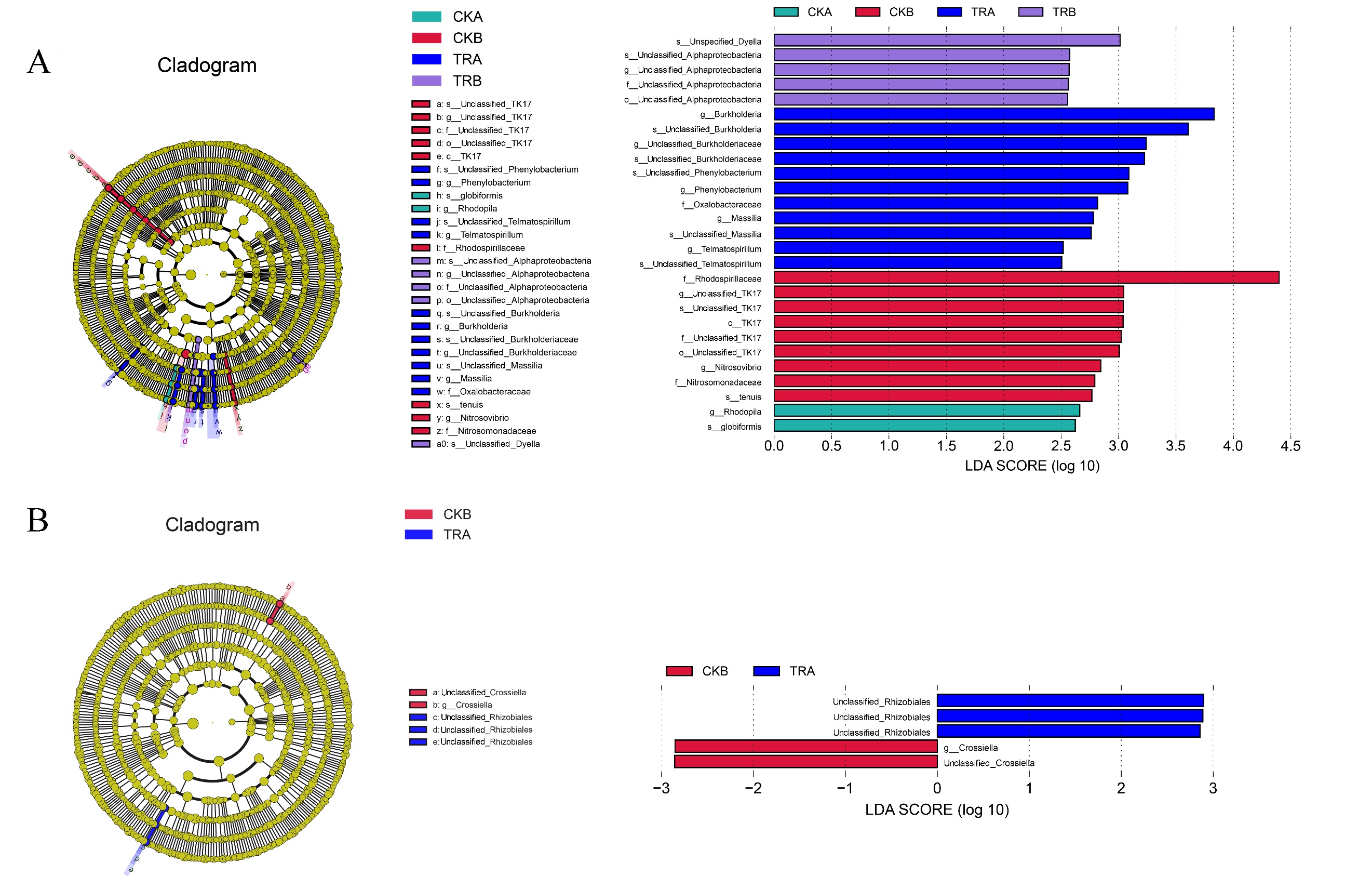
**

**Supplementary Figure 4.** Differential analysis of bacterial community in the dry (A) and rainy (B) seasons. CKA, control with 0–20 cm soil depth; CKB, control with 20–40 cm soil depth; TRA: throughfall reduction with 0–20 cm soil depth; TRB, throughfall reduction with 20–40 cm soil depth. Note: Fig S4A has been published in our previous study (Lin et al., 2022).

**
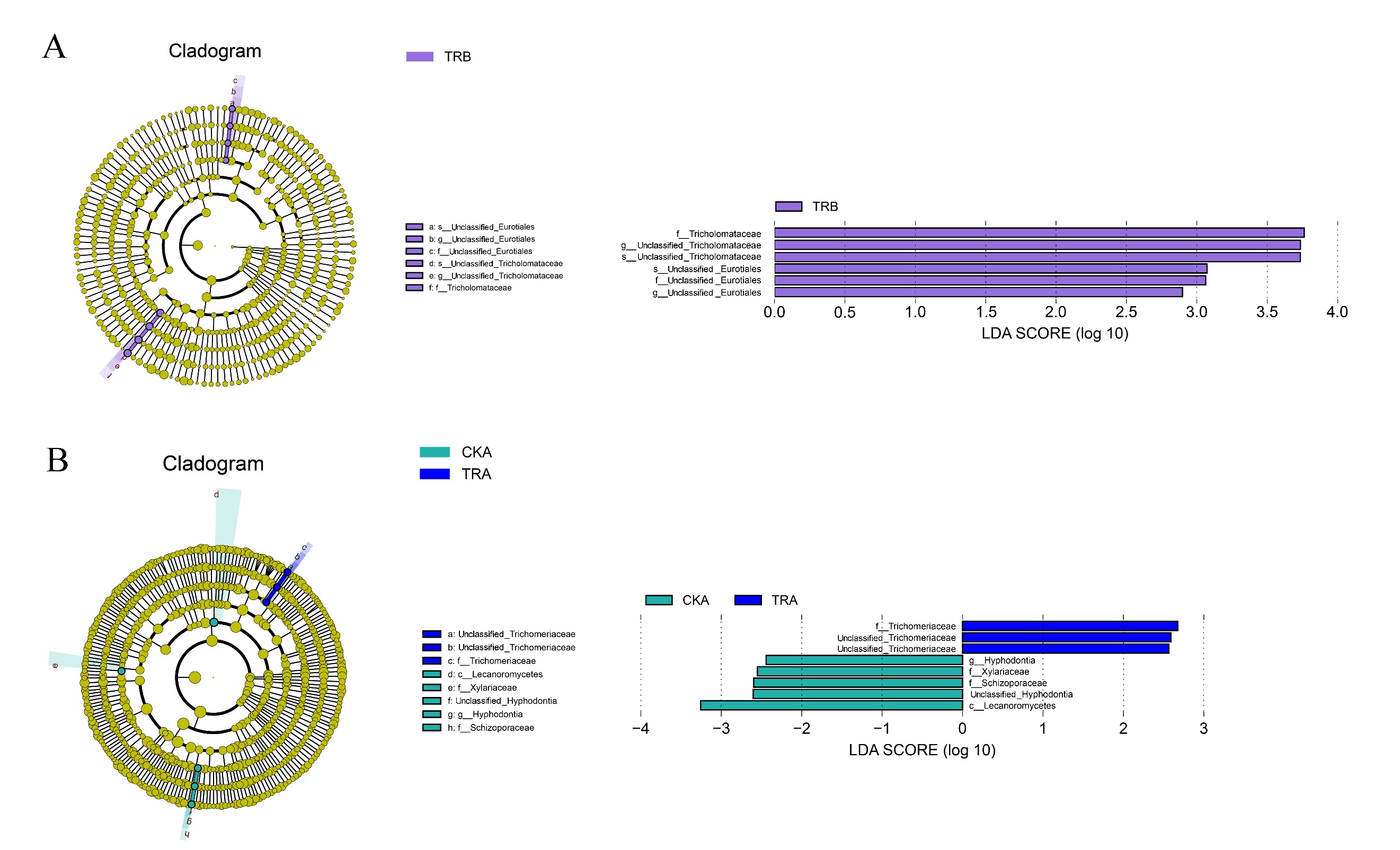
**

**Supplementary Figure 5.** Differential analysis of fungal community in the dry (A) and rainy (B) seasons. CKA, control with 0–20 cm soil depth; CKB, control with 20–40 cm soil depth; TRA: throughfall reduction with 0–20 cm soil depth; TRB, throughfall reduction with 20–40 cm soil depth. Note: Fig. S5A has been published in our previous study (Lin et al., 2022).

**
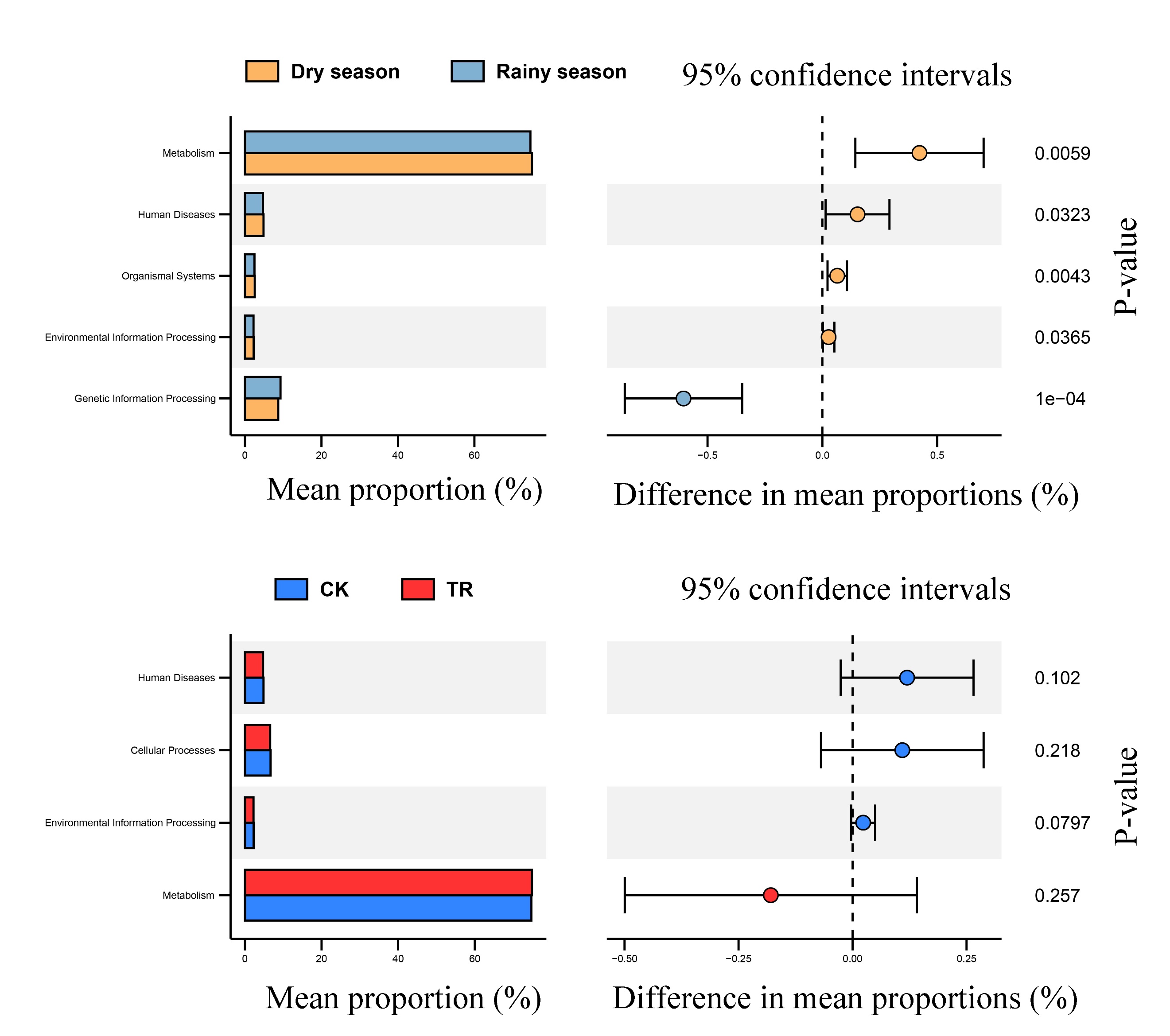
**

**Supplementary Figure 6.** STAMP analysis of bacterial function prediction of bacterial function prediction (hierarchy level 1). In the figure, Y-axis is the pathway predicted by function. Column length is the average relative abundance of the pathway in each sample group. CK: control; TR: throughfall reduction treatment.


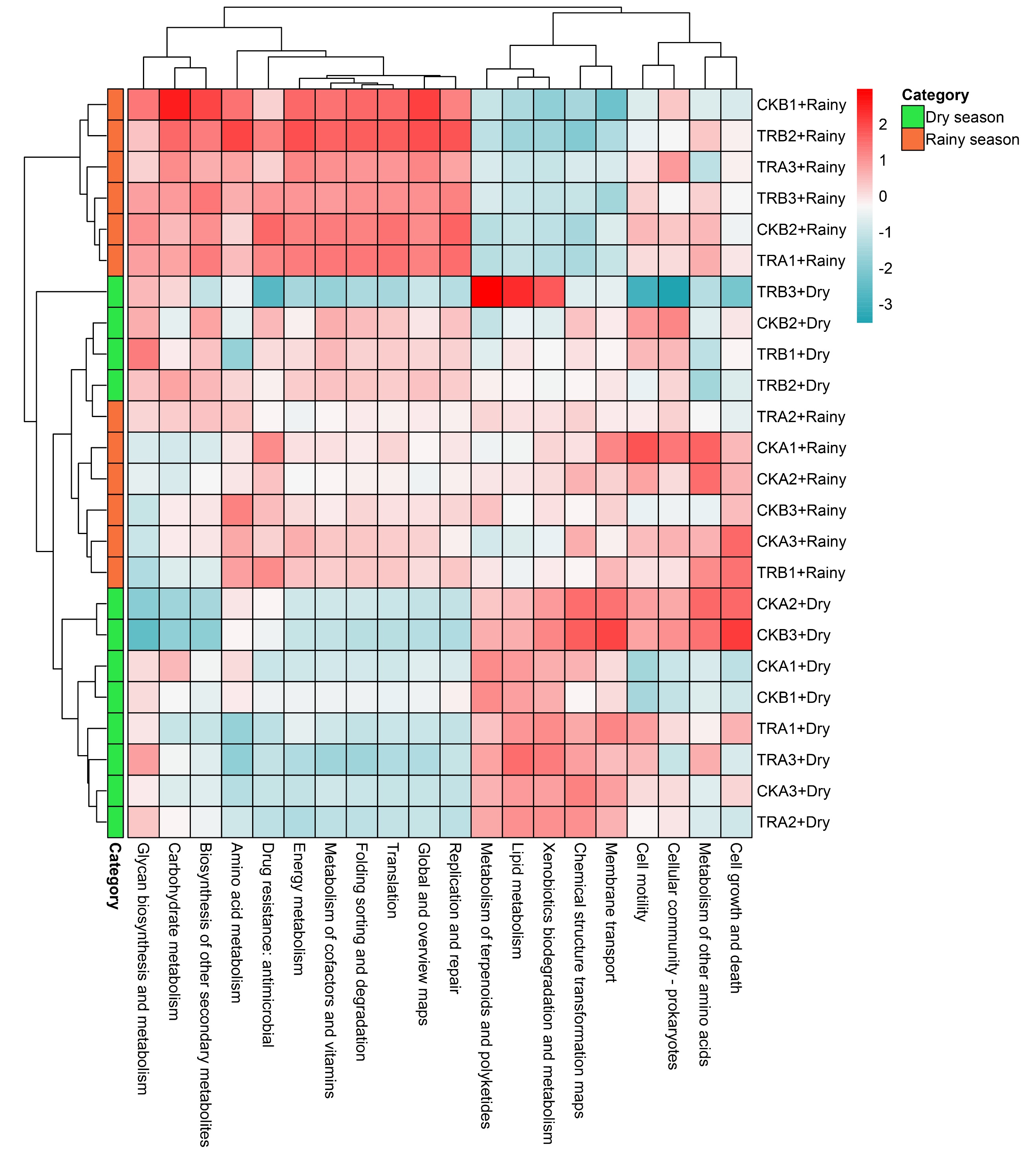


**Supplementary Figure 7.** Heat map of PICRUSt2 based functional predicted for bacterial of different samples (hierarchy level 2). Only the top 20 predicted functions in terms of relative abundance were shown. CKA, control with 0–20 cm soil depth; CKB, control with 20–40 cm soil depth; TRA: throughfall reduction with 0–20 cm soil depth; TRB, throughfall reduction with 20–40 cm soil depth. CKA1, CKA2, and CKA3 represent three replicates of CKA, and so on. Dry: the dry season; Rainy: the rainy season.

**Supplementary Table 1**. The result of envfit function of R packages indicated correlation of each soil physicochemical property with overall bacterial communities for TR (throughfall reduction) and CK (control).

| **Soil depth** | **Index** | **RDA1** | **RDA2** | **R^2^** | **Pr (>r)** |
| --- | --- | --- | --- | --- | --- |
| 0–20 cm | TN | -0.978783 | -0.2049 | 0.2528388 | 0.2573713 |
|  | TP | -0.991173 | -0.132575 | 0.6183212 | **0.0084958** |
|  | AHN | -0.993785 | 0.1113152 | 0.8141242 | **0.0004998** |
|  | AP | -0.007395 | -0.999973 | 0.4813537 | 0.0514743 |
|  | SWC | 0.6740202 | -0.738713 | 0.0439783 | 0.8130935 |
|  | TOC | -0.971212 | 0.238217 | 0.6498109 | **0.0069965** |
|  | TK | -0.191763 | 0.9814412 | 0.188511 | 0.3963018 |
|  | pH | -0.962891 | 0.26989 | 0.687473 | **0.003998** |
|  | C/N ratio | -0.828163 | 0.560487 | 0.5622923 | **0.0209895** |
|  | C/P ratio | 0.0438225 | 0.9990393 | 0.1050629 | 0.6256872 |
|  | N/P ratio | 0.9976519 | -0.068488 | 0.151222 | 0.4852574 |
| 20–40 cm | TN | -0.18294 | -0.98312 | 0.038396 | 0.828086 |
|  | TP | -0.92171 | 0.387876 | 0.686519 | **0.006497** |
|  | AHN | -0.70504 | 0.709163 | 0.82715 | **0.001499** |
|  | AP | -0.97032 | 0.241821 | 0.405508 | 0.088956 |
|  | SWC | 0.999261 | 0.038435 | 0.059928 | 0.772614 |
|  | TOC | -0.83603 | 0.548685 | 0.78619 | **0.002499** |
|  | TK | -0.99113 | 0.132887 | 0.193231 | 0.368816 |
|  | pH | -0.71602 | 0.698079 | 0.532626 | **0.038981** |
|  | C/N ratio | -0.82403 | 0.566549 | 0.87204 | **0.001999** |
|  | C/P ratio | -0.69216 | 0.72174 | 0.07158 | 0.723138 |
|  | N/P ratio | 0.757756 | -0.65254 | 0.425907 | 0.081959 |

Notes: RDA1 and RDA2 are the first two representative components of overall fungal community. The Pr value represent the significance of the correlation. TN, total nitrogen. TP, total phosphorus. TK, total potassium. TOC, total organic carbon. AP, available phosphorus. SWC, soil water content. AHN, alkali hydrolyzed nitrogen. C/N ratio, the ratio of total organic carbon and total nitrogen. C/P ratio, the ratio of total organic carbon and total phosphorus. N/P ratio, the ratio of total nitrogen and total phosphorus.

**Supplementary Table 2**. The result of envfit function of R packages indicated correlation of each soil physicochemical property with overall fungal communities for TR (throughfall reduction) and CK (control).

| **Soil depth** | **Index** | **RDA1** | **RDA2** | **R^2^** | **Pr (>r)** |
| --- | --- | --- | --- | --- | --- |
| 0–20 cm | TN | -0.89411 | -0.44785 | 0.348244 | 0.152424 |
|  | TP | -0.97143 | 0.237343 | 0.413753 | 0.089955 |
|  | AHN | -0.96815 | 0.250385 | 0.482588 | **0.042479** |
|  | AP | 0.978077 | -0.20825 | 0.006375 | 0.975012 |
|  | SWC | -0.7479 | -0.66381 | 0.720476 | **0.004998** |
|  | TOC | -0.99554 | 0.094336 | 0.5493 | **0.02099** |
|  | TK | -0.18032 | -0.98361 | 0.19378 | 0.381309 |
|  | pH | -0.89178 | 0.452473 | 0.577031 | **0.014993** |
|  | C/N ratio | -0.72798 | 0.685595 | 0.408766 | 0.093453 |
|  | C/P ratio | -0.62214 | -0.78291 | 0.035332 | 0.826087 |
|  | N/P ratio | 0.589554 | -0.80773 | 0.205627 | 0.370815 |
| 20–40 cm | TN | 0.447572 | -0.89425 | 0.316757 | 0.166917 |
|  | TP | -0.9782 | 0.207656 | 0.601633 | **0.012494** |
|  | AHN | -0.99422 | 0.107388 | 0.644881 | **0.008996** |
|  | AP | -0.97772 | -0.20989 | 0.151215 | 0.482759 |
|  | SWC | 0.838381 | -0.54508 | 0.041168 | 0.795602 |
|  | TOC | -0.97958 | -0.20105 | 0.678303 | **0.003998** |
|  | TK | -0.85116 | -0.52491 | 0.058766 | 0.749625 |
|  | pH | -0.94614 | 0.323753 | 0.620337 | **0.008496** |
|  | C/N ratio | -0.99974 | 0.022843 | 0.85618 | **0.0005** |
|  | C/P ratio | -0.49106 | -0.87113 | 0.197449 | 0.363818 |
|  | N/P ratio | 0.929418 | -0.36903 | 0.503261 | **0.04098** |

Notes: RDA1 and RDA2 are the first two representative components of overall fungal community. The Pr value represent the significance of the correlation. TN, total nitrogen. TP, total phosphorus. TK, total potassium. TOC, total organic carbon. AP, available phosphorus. SWC, soil water content. AHN, alkali hydrolyzed nitrogen. C/N ratio, the ratio of total organic carbon and total nitrogen. C/P ratio, the ratio of total organic carbon and total phosphorus. N/P ratio, the ratio of total nitrogen and total phosphorus.

**Supplementary Table 3** Major properties of bacterial and fungal networks as affected by CK (control) and TR (throughfall reduction) in dry and rainy seasons, and the associated random networks.

|  | Season | Treatments | Nodes/Edges | Avg. Degree | Avg.  path length | Diameter | Clust.  Coeff |
| --- | --- | --- | --- | --- | --- | --- | --- |
| Bacteria | Dry season | CK | 500/1326 | 5.304 | 1.811 | 8 | 0.937 |
|  |  | TR | 500/1413 | 5.652 | 3.77 | 12 | 0.92 |
|  | Rainy season | CK | 500/855 | 3.42 | 1 | 1 | 0.968 |
|  |  | TR | 500/785 | 3.14 | 1 | 1 | 0.97 |
| Fungi | Dry season | CK | 100/170 | 3.4 | 1.103 | 2 | 0.913 |
|  |  | TR | 100/144 | 2.88 | 1 | 1 | 0.854 |
|  | Rainy season | CK | 100/158 | 3.16 | 1 | 1 | 0.908 |
|  |  | TR | 100/168 | 3.36 | 1 | 1 | 0.844 |

Notes: Average degree is the number of edges on each node, representing how many other nodes (ASVs) in the network are connected with the given node. Path length represents the nearest distance between two nodes. Diameter is the largest distance between two nodes in a network. Clustering coefficient shows the extent a node is connected to its neighbors.
